# *In vitro* pharmacokinetic/pharmacodynamic modeling of the effect of mucin on polymyxin B activity against *Acinetobacter baumannii*

**DOI:** 10.1128/aac.01535-24

**Published:** 2025-03-26

**Authors:** Mathilde Lacroix, Jérémy Moreau, Claudia Zampaloni, Caterina Bissantz, Hélène Mirfendereski, Hamasseh Shirvani, Sandrine Marchand, William Couet, Alexia Chauzy

**Affiliations:** 1 Université de Poitiers INSERM, PHAR2 27077 , Poitiers, Nouvelle-Aquitaine, France; 2 Institut Roche–Boulogne-Billancourt, Boulogne-Billancourt, France; 3 Roche Pharma Research and Early Development, Immunology, Infectious Disease and Ophthalmology 30259 , Basel, Basel-Stadt, Switzerland; 4 Roche Pharma Research and Early Development, Pharmaceutical Sciences, Roche Innovation Center Basel, F. Hoffmann-La Roche Ltd 30259 , Basel, Basel-Stadt, Switzerland; 5 Département de Pharmacocinétique et Toxicologie, CHU Poitiers 36655 , Poitiers, Nouvelle-Aquitaine, France; University of Pittsburgh School of Medicine, Pittsburgh, Pennsylvania, USA

**Keywords:** *A. baumannii*, polymyxin B, semi-mechanistic PK/PD modeling, mucin

## Abstract

The antibacterial efficacy of polymyxins in lungs may be impacted by mucin. The aim of this study was to characterize *in vitro* the effect of mucin on polymyxin B (PMB) activity against two multidrug-resistant *Acinetobacter baumannii* strains isolated from a patient before (AB121-D0) and after colistin treatment (AB122-D12), using a pharmacokinetic/pharmacodynamic (PK/PD) modeling approach. PMB binding to mucin was characterized by ultracentrifugation in cation-adjusted Mueller-Hinton broth (CAMHB) supplemented with 1% mucin. Time-kill (TK) experiments were performed in CAMHB, with 1% mucin or without as control, and with PMB total concentrations ranging from 0.25 to 512 mg/L based on the strain’s minimum inhibitory concentration (MIC). For each strain, TK data were modeled based on unbound PMB concentrations. Bacterial resistance to PMB was investigated via MIC and whole genome sequencing from bacteria that regrew in the presence of antibiotics at the end of the TK experiments. PMB unbound fraction increased nonlinearly from 6% to 60% when total concentration increased from 0.5 to 512 mg/L. In addition to binding to PMB, mucin had an impact on PMB activity, which differed between the two strains. For AB121-D0, PMB activity increased in the presence of mucin resulting in a reduction of the bacterial regrowth, whereas for AB122-D12, a decrease in PMB activity was observed. Mutations in genes involved in PMB resistance appeared randomly and explained only partially the bacterial regrowth observed in TK with antibiotics. This study showed that PMB binding to mucin had a real and important impact but was not the only factor explaining the impaired PMB efficacy in the presence of mucin.

## INTRODUCTION

*Acinetobacter baumannii* is a critical pathogen for patients with hospital-acquired pneumonia that is related to high morbidity and mortality (1). Treating these infections can be extremely difficult due to the high prevalence of multidrug-resistant (MDR) *A. baumannii* strains (2). First left behind due to their nephrotoxic properties (3), polymyxins (colistin and polymyxin B [PMB]) are now used as last-resort antibiotics, as they show preserved *in vitro* activity against MDR *A. baumannii* (4, 5). However, to reach the bacteria in infected lungs, polymyxin antibiotics need to diffuse through the mucus layer where they may bind to mucus particles, impairing their availability at the site of infection (6). Mucus is composed of 90–95% of water and 1–2% of lipids, but its key component is mucin that corresponds to 1–5% of its composition in physiological condition (7). Mucin molecules are composed mainly of carbohydrates and proteins, assembled together to form a multimeric network, responsible for the viscoelasticity properties of mucus. Gel-forming mucin has a protective role and serves also for lubrication (8), but it can also delay the antibiotic diffusion through the mucus layer and bind to certain antibiotics such as polymyxins, aminoglycosides, and fluoroquinolones due to its polymeric composition and overall negative charge (9–12). In the airways, mucin binding may lead to insufficient unbound antibiotic concentration at the site of infection, reducing antibacterial efficacy. In particular, colistin and PMB showed a strong binding to mucin, explaining in part the >100-fold increase in minimum inhibitory concentrations (MICs) in the presence of mucin for multiple gram-negative bacteria including *A. baumannii* (13). However, this previous study evaluating PMB binding to mucin by equilibrium dialysis has some limitations, including an insufficient incubation time (4 h) to reach equilibrium and the lack of assessment of nonspecific binding to the dialysis equipment, which may have led to inaccurate determination of the PMB unbound fraction. Moreover, to the best of our knowledge, the efficacy of PMB in the presence of mucin was only assessed at a single time point, by determining the MIC after 24 h of incubation (13) or the number of surviving bacteria after 2 h of exposure to the antibiotic (12), which did not allow for properly characterizing the impact of mucin on the time course of the antibacterial effect.

The aim of this study was to assess in more detail the impact of mucin on the *in vitro* activity of PMB against *A. baumannii*, using a semimechanistic pharmacokinetic/pharmacodynamic (PK/PD) modeling approach.

## RESULTS

### PMB binding to mucin

**Fig 1:**
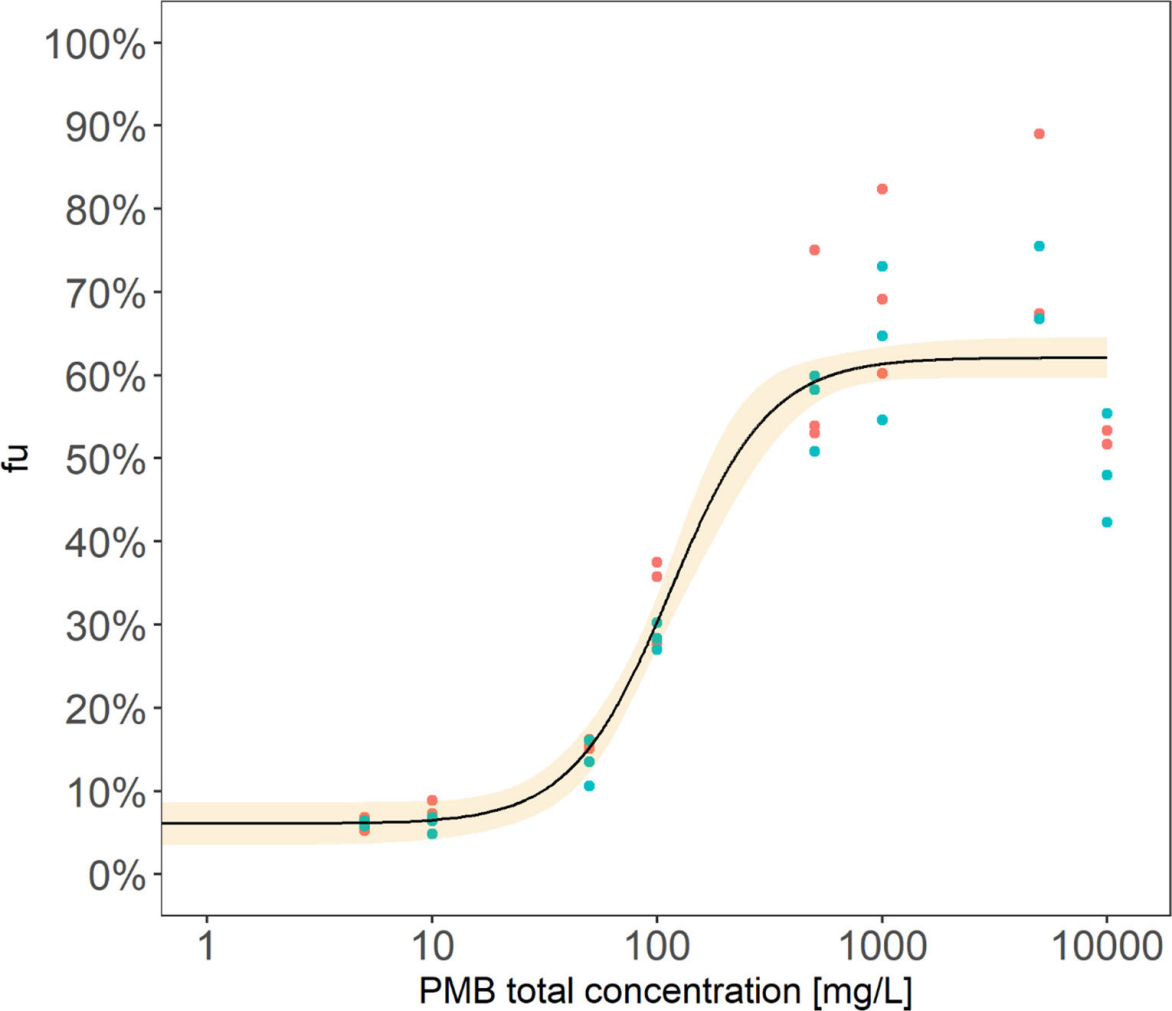
Relationship between PMB unbound fraction (*f*u) (%) and PMB total concentration (mg/L). Red and turquoise dots correspond to *f*u values calculated as the ratios of PMB unbound to total concentrations in the presence of 1% autoclaved and nonautoclaved mucin, respectively. The solid line represents the fit of the sigmoidal Emax function (equation 1, *R*² =0.92) and the yellow-colored area the 90% CI.

PMB unbound fraction determined in cation-adjusted Mueller-Hinton broth (CAMHB) with 1% mucin remained relatively stable at 6% for PMB concentrations up to 20 mg/L, then increased nonlinearly up to 60% at a PMB concentration of 200 mg/L. (Fig. 1). Parameters of equation 1, estimated either from data generated with autoclaved mucin or from data generated without autoclave, were not significantly different from those estimated from all pooled data (*P* value = 0.26–4 DDL), resulting in very similar *f*u values regardless of how the mucin solution was prepared (Table S1 and S2). Consequently, the final parameters describing the relationship between *f*u and total PMB concentration were estimated using all data pooled into a single data set (Table 1).

**TABLE 1:** Parameter estimates of the Emax model describing the relationship between *f*u and PMB total concentrations in the presence of mucin

Parameter	Description (Unit)	Value (Variance)
E0	Minimum PMB *fu* value	0.061 (0.0004)
Imax	Maximum PMB *fu* increase	0.56 (0.0009)
IC_50_	PMB total concentration needed to reach 50% of the maximum *fu* (mg/L)	114.37 (196)
ε	Hill coefficient	1.97 (0.252)

### Mucin effect on MIC

For AB121-D0, PMB MIC was increased 64-fold in the presence of 1% mucin (64 mg/L) compared to the MIC in CAMHB (1 mg/L) (Table 2). For AB122-D12, the MIC was 32 mg/L in CAMHB and 1024 mg/L in CAMHB with 1% mucin, showing a 32-fold increase (Table 2). MIC values obtained in the presence of mucin were corrected by multiplying them by *f*u in order to express them as unbound concentrations, that is, unbound MICs (MIC_u_), whereas MICs in the absence of mucin corresponded directly to MIC_u_ values. The MIC_u_ ratio in the presence and absence of mucin was then equal to 13 for AB121-D0 and 20 for AB122-D12 (Table 2).

**TABLE 2:** MIC values of PMB in CAMHB supplemented or not with 1% mucin [mg/L]

MIC (mg/L)	CAMHB	CAMHB + 1% mucin	CAMHB + 1% mucin MIC_u_ (corrected by *f*u)
*AB121-D0*	1	64	13
*AB122-D12*	32	1024	628

### Time-kill (TK) experiment and PK/PD modeling

Regrowth was observed in TK after an initial CFU decay for PMB concentrations up to 8 mg/L for AB121-D0 and 128 mg/L for AB122-D12 in CAMHB (Fig. S1). In the presence of mucin and after correction of total concentrations by *f*u, the CFU versus time profiles of the two strains were shown to be different from those observed in the absence of mucin for similar unbound PMB concentrations (Fig. S1). Regrowth was observed for AB121-D0 for unbound PMB concentrations ≤0.5 mg/L for two-thirds of replicates and ≤13 mg/L for one-third of replicates. For AB122-D12, no PMB concentration in the range tested (up to 304 mg/L) was able to prevent bacterial regrowth in the presence of mucin.

**Fig 2:**
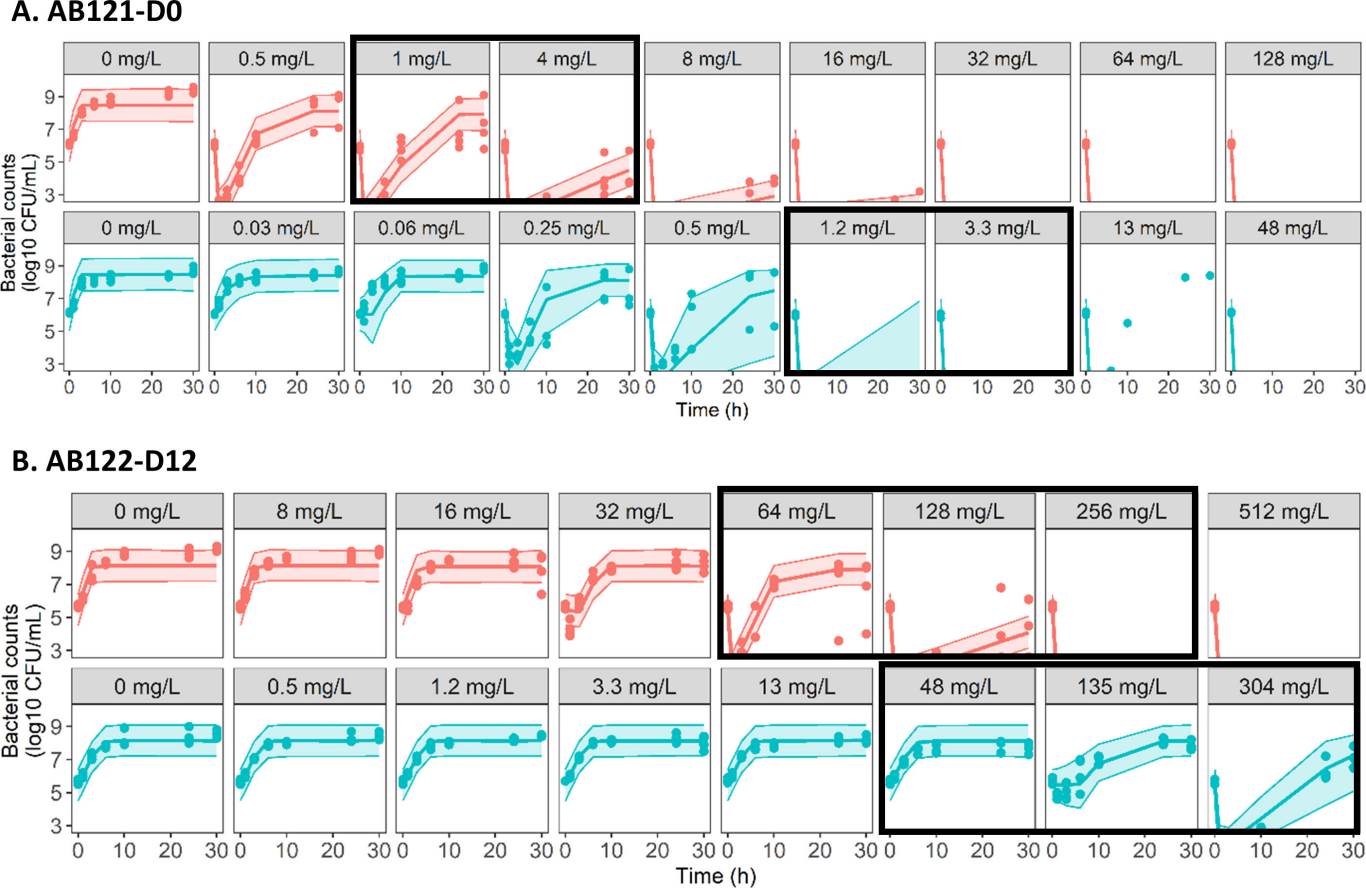
Visual predictive checks of the final models for AB121-D0 (A) and AB122-D12 (B). Circles represent experimental data, solid lines depict the median of simulated data, and colored areas depict the 90% prediction interval for 1,000 simulated profiles. Red: data without mucin. Turquoise: data with 1% mucin. The PMB concentrations indicated represent PMB unbound concentrations in TK experiments. Black boxes highlight different CFU profiles observed with and without mucin for similar concentrations of unbound PMB.

In the absence of mucin, the killing effect of PMB was characterized by an Emax model for AB121-D0 and a sigmoidal Emax model for AB122-D12 (Fig. S2, equations S5–S8), with the maximum PMB kill rate constant (Emax) reduced by around 90% for the R subpopulation compared with the S subpopulation of both strains in CAMHB (Table 3–w/o mucin). TK data in the presence of mucin were then included in the data set. When only the total PMB concentrations were corrected for mucin binding by *f*u, then the model predicted AB121-D0 regrowth at unbound PMB concentrations of 1.2 and 3.3 mg/L, whereas this was not observed experimentally (Fig. S3.1, black boxes). In contrast, for AB122-D12, the regrowth observed at unbound PMB concentrations of 135 and 304 mg/L in the presence of mucin was not captured by the model (Fig. S3.2, black boxes). Thus, in order to improve the fit, an additional mucin effect was added as a categorical covariate on the maximal PMB effect parameter for R subpopulation (Emax_R_) for AB121-D0 (∆objective function value [OFV] = 152) and on K_net_ and EC_50_ for AB122-D12 (∆OFV = 307) (Fig. S2). For AB121-D0, Emax_R_ increased by 1.4-fold in the presence of mucin. For AB122-D12, K_net_ was divided by approx. twofold and EC_50_ was increased approx. fivefold (Table 3–with mucin). The final PK/PD models adequately described the TK data, as shown on goodness-of-fit (GOF) plots available in supplemental materials (Fig. S4) and on visual predictive checks (VPCs) in Fig. 2.

**TABLE 3:** PK/PD model parameter estimates for AB121-D0 and AB122-D12^*a*^

Parameter	Description (Unit)	Estimate (95% CI)			
		AB121-D0		AB122-D12	
		Without mucin	With mucin	Without mucin	With mucin
INOC	Initial inoculum (log10 CFU/mL)	6.0 (5.89 to 6.11)		5.45 (5.33 to 5.56)	
BMAX	Maximum bacterial population size (log10 CFU/mL)	8.47 (8.35 to 8.60)		8.14 (8.05 to 8.23)	
MUT	Proportion of resistant bacteria in the initial inoculum (log10)	−4.72 (−5.01 to −4.42)		−4.19 (−4.58 to −3.84)	
K_net_	Apparent growth rate constant (h^−1^)	2.83 (2.72 to 2.93)		2.42 (2.18 to 2.70)	1.34 (1.15 to 1.52)
Emax_S_	Maximum rate constant of PMB effect on susceptible subpopulation (h^−1^)	21.1 (19.03 to 23.77)		33.4 (26.9 to 40.4)	
Emax_R_	Maximum rate constant of PMB effect on resistant subpopulation (h^−1^)	2.84 (2.74 to 2.94)	3.90 (3.59 to 4.20)	2.61 (2.32 to 2.92)	
EC_50_	PMB unbound concentration needed to reach 50% of Emax for both S & R subpopulations (mg/L)	0.40 (0.36 to 0.45)		73.3 (67.3 to 79.5)	384 (342 to 437)
γ	Hill coefficient on PMB effect	/^*b*^		3.03 (2.64 to 3.48)	
σ	Additive residual error on log10 scale for total bacteria count (log10 CFU/mL)	0.35 (0.29 to 0.41)		0.33 (0.28 to 0.39)	

^*a*^ CI: Confidence Interval; PMB: polymyxin B. The 95% CI was obtained by Sampling Importance Resampling (SIR).

^*b*^ "/": the gamma parameter was not included in the model for strain AB121-D0.

### Characterization of bacterial regrowth

For AB121-D0 in the absence of mucin, MIC values increased (≥32-fold) for 6 out of 10 (60%) bacterial samples collected at T30h in TK experiments after bacterial regrowth compared to control MICs determined at T30h in the absence of antibiotic (Table 4). In the presence of mucin, an increase of MICs of the same order of magnitude was observed for 5 samples out of 16 (31%) (Table 5). Mutations in *pmrABC*, corresponding to single nucleotide mutations, duplication, or transposon insertion into the promoter, were detected in 9 out of 11 samples (82%) for which the PMB MIC was increased at the end of TK experiments (Table 5).

**TABLE 4:** MIC values (mg/L) determined at the end (T30h) of TK experiments conducted in CAMHB^*b*^

Parental strain	PMB concentrations in TK tubes from which bacteria were sampled	TK replicate 1	TK replicate 2	TK replicate 3	TK replicate 4
AB121-D0	Control^*a*^ (0 mg/L)	1	0.5	0.25	0.25
	0.5 mg/L	0.5	**>16**	**8**	**>16**
	1 mg/L	1	0.5	0.25	**8**
	4 mg/L	**>16**	**>16**		
AB122-D12	Control^*a*^ (0 mg/L)	64	256	64	128
	8 mg/L	64	32	64	64
	16 mg/L	**256**	64	64	256
	32 mg/L	64	256	64	128
	64 mg/L	128	128	64	
	128 mg/L	128	64	**256**	

^*a*^ Control MICs were determined at T30h of TK experiments in the absence of antibiotic.

^*b*^ In bold are the MIC values that are increased by a factor of ≥ 4 compared to the control MIC. Gray boxes correspond to replicates for which the MIC could not be determined at 30h because no regrowth was observed.

**TABLE 5:** MIC values (mg/L) determined at the end (T30h) of TK experiments conducted in CAMHB with 1% mucin^*b*^

Parental strain	PMB unbound concentrations in TK tubes from which bacteria were sampled	TK replicate 1	TK replicate 2	TK replicate 3	TK replicate 4
AB121-D0	Control^*a*^ (0 mg/L)	0.5	0.5	0.25	0.25
	0.03 mg/L	0.5	1	0.5	0.25
	0.06 mg/L	0.5	0.25	0.5	0.25
	0.25 mg/L	0.5	0.25	**>16**	**8**
	0.5 mg/L		**>16**	**>16**	0.25
	13 mg/L				**>16**
AB122-D12	Control^*a*^ (0 mg/L)	64	128	64	128
	0.5 mg/L	64	128	64	64
	1.2 mg/L	64	128	64	128
	3.3 mg/L	64	64	64	256
	13 mg/L	64	256	128	**>512**
	48 mg/L	64	**512**	64	256
	135 mg/L	**256**	256	**512**	**512**
	304 mg/L			128	256

^*a*^ Control MICs were determined at T30h of TK experiments in the absence of antibiotic.

^*b*^ In bold are the MIC values that are increased by a factor of ≥ 4 compared to the control MIC. Gray boxes correspond to replicates for which the MIC could not be determined at 30 h because no regrowth was observed.

For AB122-D12, a ≥fourfold increase in MIC compared with control values was observed for 11% (2/18) and 19% (5/26) of samples taken after bacterial regrowth in TK experiments in the absence and presence of mucin, respectively (Tables 4 and 5). Mutations in *lpxACD* genes in addition to the 10 amino acid insertion in *pmrB* were detected in four of the seven samples (57%) for which the PMB MIC was increased at the end of TK experiments (Table 6). For two samples (128 mg/L replicates 1 and 2), two mutations in *lpxA* were found, with no significant elevation of MIC values compared to control.

**TABLE 6:** Relevant mutations detected from AB121-D0 and AB122-D12 at the end of TK experiments

Parental strain	Medium in TK tubes from which bacteria were sampled	PMB concentrations in TK tubes from which bacteria were sampled	TK replicate N°	MIC fold-change^*a*^	Mutation type		Mutated gene	Protein associated
AB121-D0	CAMHB	0.5 mg/L	2	>32	Arg263His	Missense	*pmrB*	Signal transduction histidine kinase
			3	32	ISAba1 insertion in the promoter	Insertion	*pmrC_3*	Phosphoethanolamine—lipid A transferase
			4	>64	Asn353Tyr	Missense	*pmrB*	Signal transduction histidine kinase
		1 mg/L	4	32	16.25dup	Duplication	*pmrB*	Signal transduction histidine kinase
		4 mg/L	2	>32	Pro170Leu	Missense	*pmrB*	Signal transduction histidine kinase
	CAMHB + 1% mucin	0.25 mg/L	3	>64	Phe164Ile	Missense	*pmrB*	Signal transduction histidine kinase
		0.5 mg/L	2	>32	Pro170Leu	Missense	*pmrB*	Signal transduction histidine kinase
			3	>64	Arg231Leu	Missense	*pmrB*	Signal transduction histidine kinase
		13 mg/L	4	>64	Ala227Val	Missense	*pmrB*	Signal transduction histidine kinase
AB122-D12	CAMHB	16 mg/L	1	4	Arg258Leu	Missense	*lpxA*	Acyl-ACP--UDP-N-acetylglucosamine O-acyltransferase
		128 mg/L	1	2	Trp206Arg Thr210Ala	Missense	*lpxA*	UDP-N-acetylglucosamine acyltransferase
			2	0.25	Trp206Arg Thr210Ala	Missense	*lpxA*	UDP-N-acetylglucosamine acyltransferase
	CAMHB + 1% mucin	48 mg/L	2	4	Ser224Pro	Missense	*lpxA*	UDP-N-acetylglucosamine acyltransferase
		135 mg/L	3	8	ISAba13 insertion in the promoter	Insertion	*lpxC*	UDP-3-O-acyl-N-acetylglucosamine deacetylase
			4	4	ISAba13 insertion in the promoter	Insertion	*lpxC*	UDP-3-O-acyl-N-acetylglucosamine deacetylase

^*a*^ MIC fold-change were calculated regarding control value of respective replicate.

## DISCUSSION

This study has shown that PMB binding to mucin is saturable, which could not be observed in previous studies performed at a single PMB concentration (12, 13). The initially low *f*u (~6%) for total PMB concentrations up to 20 mg/L increased then nonlinearly with concentrations, reaching a maximum value of 60% for concentrations higher than 200 mg/L. Therefore, for PMB concentrations encountered in clinical practice, up to 10 mg/L (14), approximately 95% of PMB would be bound to mucin. A single percentage of mucin was evaluated in this study, chosen to reflect the physiological composition of mucus in patients with mild cystic fibrosis (15, 16). However, this percentage could be increased in case of severe chronic lung diseases such as cystic fibrosis with pulmonary exacerbation, chronic obstructive pulmonary disease, or cancer (15, 17–19). In addition, mucin represents only one of the components of mucus, and additional components such as lipids or DNA might also contribute to drug binding *in vivo*, as observed previously for other cationic compounds (6, 12, 20). Therefore, the PMB binding measured in this study may be underestimated compared to what actually occurs in patients.

The binding determination for a wide range of PMB concentrations, largely exceeding those encountered clinically, allowed us to correct the MICs and the concentrations tested in TK experiments (ranging from 0.5 to 1024 mg/L) by multiplying them by the appropriate *f*u value and therefore to express all concentrations as unbound and thus active concentrations.

The impact of mucin on PMB activity was first assessed by determining unbound MICs (MIC_u_) in the absence and presence of mucin (*i.e*. corrected by *f*u in the latter case). We observed that MIC_u_ values in the absence and presence of mucin were increased by a factor of 13 (from 1 to 13 mg/L) for AB121-D0 and by a factor of 20 (from 32 to 628 mg/L) for AB122-D12, suggesting that in addition to PMB binding, mucin may inhibit PMB antimicrobial activity against these two *A. baumannii* strains, as previously observed with fluoroquinolones (12) which, despite 100% *f*u, showed reduced activity against *P. aeruginosa* in the presence of mucin.

To better characterize the impact of mucin over time, TK experiments were performed, and data were modeled using a semimechanistic PK/PD approach. In the absence of mucin, for both strains, the biphasic behavior with an initial CFU decay followed by regrowth, commonly observed for gram-negative bacteria, was properly described by a model with heteroresistance (S/R). Mucin, in addition to binding to PMB, had an impact that differed between strains by affecting different model parameters and, above all, by affecting PMB activity in opposite directions. For AB121-D0, an increase in PMB activity was observed in the presence of mucin with a reduction of the apparent bacterial regrowth, explained by an increase in Emax for the R subpopulation by a factor of 1.4 between the values in the absence and presence of mucin. For AB122-D12, a decrease in the initial CFU decay with time as well as an increase in the bacterial regrowth were observed in the presence of mucin and characterized by a fivefold increase in EC_50_ for both S and R subpopulations. Furthermore, an effect of mucin on the growth rate of AB122-D12, in addition to its impact on PMB activity, was observed with a twofold decrease between the values of K_net_ in the absence and presence of mucin.

To better understand how mucin influenced resistance mechanisms responsible for bacterial regrowth in the presence of PMB for both strains, MIC determination and sequencing were conducted at the end of TK experiments for both conditions—with and without mucin. Bacterial regrowth observed after an initial CFU decay in TK experiments was associated with an increase in the MIC at T30h in 42% and 16% of cases for AB121-D0 and AB122-D12, respectively, with variability appearing between replicates for the same PMB concentration tested. These MIC increases were often explained by the appearance of mutations (9 times out of 11 for AB121-D0 and 4 times out of 7 for AB122-D12) in different genes, depending on the strain. Overall, no specific impact of mucin was observed on MIC changes or the occurrence of mutations. When identified, mutations in AB121-D0 corresponded always to modifications of the *pmrABC* operon, known to confer PMB resistance through constitutive *eptA* expression, thus reducing the overall net-negative charge of the membrane by lipid A modification (21). Although different from the mutation that gave AB122-D12 its initial resistance to PMB (p.21_22insCILIFSVILG), these mutations occurred in the same operon. For AB122-D12, sequencing revealed mutations inducing inhibition of LOS synthesis by the impairment of *lpxACD,* thus preventing PMB binding to the bacterial membrane (22). Therefore, in addition to differences between strains, this study highlighted intra-strain (i.e., between experiments) differences with mutations occurring randomly and varying from replicate to replicate, while no differences were observed on CFU versus time profiles. The identification of mutations in some but not all samples collected after regrowth suggested the involvement of other resistance mechanisms, complicating the semimechanistic PK/PD modeling of the data. To further assess the mechanistic significance of the model, TK data for AB121-D0 in CAMHB were modeled again after separating replicates in which a mutation conferring resistance to PMB was identified and replicates in which no mutation was observed, using different model structures (i.e., models with heteroresistance or adaptive resistance). The VPCs presented in supplemental material (Fig. S5) show that, when mutations were identified, the S/R model did not provide a better characterization of the data, and conversely, when an R subpopulation was not experimentally identified, the use of a model with adaptation did not improve the fit of the data.

This study has several limitations that may restrict the generalization of its results. First, only two strains of *A. baumannii* were tested, and the divergent effects of mucin between these strains limit the applicability of findings to other strains, as the biological basis for these differences remains to be determined. Second, the investigation of resistance mechanisms underlying bacterial regrowth observed in TK experiments was limited to sequencing. Thus, while mutations were identified in only certain experimental replicates, other potential resistance mechanisms may have been overlooked. Third, although the semimechanistic PK/PD model developed in this study adequately described the TK data and differentiated the impacts of mucin on PMB binding, bacterial growth, and killing kinetics over the experiment time course—which cannot be achieved by traditional MIC determination (23)—it is worth remembering that this model remains a simplification of biological reality. Indeed, the same model structure incorporating heteroresistance (S/R model) successfully described the TK data, showing an initial bacterial decrease followed by regrowth of different origins (mutations versus other unidentified mechanisms). This demonstrates that, despite being classified as semi-mechanistic due to the incorporation of biological knowledge, these models fail to provide a true mechanistic explanation of the experimental data. Their structure and parameter estimates are largely driven by observed data, making them far simpler than the complex reality, which probably involves multiple successive or simultaneous resistance mechanisms (24). Consequently, if the models cannot fully explain the experimental results, then changes in model parameters, such as those induced in the presence of mucin, also remain difficult to explain.

Yet, several biological hypotheses could support the study’s findings, in particular the fact that mucin does not simply have a binding effect on the antibiotic. First, various bacterial species including *A. baumannii* have demonstrated the ability to degrade mucin (25–27). This degradation may alter the conformation of mucin glycoproteins, potentially modifying the *f*u of PMB and thus the concentration-effect relationship established in this study. Furthermore, the byproducts of mucin degradation, such as carbohydrates, can serve as an energy source for bacteria, thereby influencing their energy and sugar metabolism and potentially their growth (25, 26). Moreover, mucin has been shown to modify lipid metabolism and components of the bacterial cell wall (25, 26, 28), both known to play a major role in PMB efficacy and in resistance mechanisms developed by bacteria. Additionally, the presence of mucin can significantly influence the virulence of *A. baumannii* (25, 29). As observed for other species (26, 30, 31), mucin-induced changes in virulence can lead to changes in bacteria’s shape, growth patterns, aggregation, motility, and quorum sensing. These changes can therefore potentially alter the drug efficacy.

In conclusion, this study provides valuable insights into how mucin influences *A. baumannii* responses to PMB while highlighting the need for further investigation into the underlying biological mechanisms in order to better understand the interaction between mucin, bacteria, and antibiotics. Although PK/PD models could be improved by incorporating more mechanistic knowledge, they remain a useful tool for characterizing data that are homogeneous in terms of the observed effect.

## MATERIALS AND METHODS

### Chemicals and strains

PMB was obtained from Sigma-Aldrich (Merck KGaA, Saint-Quentin Fallavier, France) and used to prepare stock solutions in sterile water.

Mucin from the porcine stomach was obtained from Sigma-Aldrich (Merck KGaA, Saint-Quentin Fallavier, France) and solubilized in NaCl 0.9% to obtain a solution at 20% (pH adjusted to 7). The solution was autoclaved at 110°C for 15 minutes.

Two clinical MDR strains of *A. baumannii*, isolated from a patient before (AB121-D0) and 12 days after treatment with colistin (AB122-D12), were used during this study (32). AB122-D12 carries a 10 amino acid insertion into the *pmrB* gene, known to confer PMB resistance.

### MIC determination

PMB MICs of both *A. baumannii* strains were determined in commercial CAMHB (BioMérieux, Marcy-l’Etoile, France) according to the European Committee on Antimicrobial Susceptibility Testing and Clinical and Laboratory Standards Institute (CLSI) recommendations (33, 34). PMB MICs of the two *A. baumannii* isolates were also determined at least in duplicate in CAMHB supplemented with 1% of mucin.

### PMB binding to mucin

PMB solutions at concentrations ranging from 0.5 to 10,000 mg/L in CAMHB with 1% mucin were incubated for 1 h at 35°C, then ultracentrifuged at ~290 000 g for 5 h at room temperature (Beckman Optima LE-80K, rotor: Beckman Coulter 70.1 Ti), according to a method previously described (35). Unbound PMB concentrations in the supernates were determined by LC-MS/MS (method adapted from a previous publication (36) and detailed in Supplemental material) and used to determine the unbound fraction (*fu*) of PMB. Potential PMB sedimentation (37) during experimentation was determined from PMB solutions prepared in mucin-free CAMHB and subjected to the same ultracentrifugation conditions as previously described, and then used to correct for unbound PMB concentrations.

The same procedure was performed with a nonautoclaved mucin solution. Experiments were done in triplicate.

The relationship between *fu* and PMB total concentrations was modeled according to a sigmoidal Emax function (equation 1):


 (1) $$fu=E0+\;\frac{Imax\;\times \;C^{\varepsilon }}{IC_{50}^{\varepsilon }+\;C^{\varepsilon }},$$

where E0 represents the minimum *fu*, Imax the maximum increase in *fu*, IC_50_ (mg/L) corresponds to PMB total concentration needed to reach 50% of Imax, C (mg/L) to the PMB total concentration, and ε to the Hill coefficient.

Parameter estimation was performed using nonlinear regression with iteratively reweighted least squares (R, version 4.1.3 – Package: robustbase, version 0.99–0). Two sets of parameters were estimated, one for the data generated with autoclaved mucin, another for the data generated without autoclave. The two sets of parameters were compared using ANOVA testing.

### TK experiments

Bacteria were cultured overnight under shaking (150–170 rpm) in CAMHB at 35°C. The bacterial suspension was then diluted to 1/50 and incubated at 35°C under shaking until an optical density of 0.3 (corresponding to 1.10^8^ CFU/ml of bacteria in the exponential growth phase) was achieved. This bacterial suspension was then diluted in order to obtain a starting inoculum at 1.10^6^ CFU/mL. TK experiments were performed in CAMHB in the absence and presence of 1% mucin. PMB concentrations varied from 0.5 mg/L to 128 mg/L for AB121-D0 and 8 mg/L to 512 mg/L for AB122-D12. Drug-free CAMHB was used as bacterial growth control for each strain. Tubes were incubated at 35°C under shaking (150–170 rpm), and aliquots were sampled over time at T0h, T1h, T3h, T6h, T10h, T24h, and T30h. Samples were serially diluted in NaCl 0.9% and plated onto Mueller-Hinton agar supplemented with 1% activated charcoal (to prevent PMB carry-over effect). Bacteria were counted after overnight incubation at 35°C. The limit of quantification was fixed based on CLSI guidelines (38) and was equal to 400 CFU/ml (*i.e*. 2.6 log_10_ CFU/mL). Experiments were conducted in triplicate for both strains.

### Characterization of bacterial regrowth

Samples of 1 mL were collected at T30h of TK experiments from tubes where regrowth was observed in the presence of antibiotics to determine stable resistance via MIC and whole genome sequencing. Samples were centrifuged (8 minutes at 8,000 rpm); then, the supernates were removed, and the pellets were stored at −80°C in CAMHB-glycerol 20%. A minimum bacterial density of ~10^5^ CFU/mL in samples collected at the end of TK experiments was required for MIC susceptibility testing and sequencing.

Prior to MIC testing, the isolates were streaked onto a drug-free Mueller-Hinton agar plate and incubated for 24 h.

DNA of bacteria sampled from tubes showing regrowth at T30h in TK experiments were, after extraction, sequenced using a MinION sequencer (Oxford Nanopore Technologies (ONT), Oxford, UK). The sequencing method is described in detail in the supplemental materials, which include also accession numbers and links of bacterial samples registered on GenBank (Table S1).

### Pharmacodynamic modeling

Bacterial counts obtained from TK experiments without mucin were first modeled using NONMEM 7.4 (ICON plc, Dublin, Ireland) with the Laplacian algorithm and the M3 method for handling observations below the limit of quantification (39). PMB was assumed to be stable over the course of the experiment as previously shown (40). A previously described model with hetero-resistance (41), that is, two bacterial subpopulations, one susceptible to PMB (S) and one resistant (R), was used to describe the time course of bacterial counts for the two *A. baumannii* strains (Fig. 1). Different functions were tested to characterize the PMB killing effect (see supplemental material for model building).

Then, TK data in the presence of mucin were included in the data set and PMB total concentrations were corrected for mucin binding using equation 1, with unbound PMB concentrations driving the antibacterial effect. Random variability between TK experiments (one experiment corresponding to a replicate of a tested concentration) was included to account for the uncertainty associated with each parameter of equation 1 and thus with the estimation of unbound PMB concentration in TK, such as:


(2) $$fu_{i}=E0_{i}+\;\frac{Imax_{i}\;\times \;C^{\varepsilon_{i}}}{IC_{50,i}^{\varepsilon_{i}}+\;C^{\varepsilon_{i}}},$$

where the index *i* represents the *i*th experiment, each parameter value being specific to a given experiment. Random variability was included via an additive function for each parameter of equation 2. For example, E0_*i*_ was defined as follows:


(3) $$E0_{i}=E0_{pop}+\eta_{i},$$

where E0_pop_ is the population parameter (i.e., fixed effect) and η_*i*_ is the deviation from the population value for the *i*th experiment. The individual η_*i*_ values were assumed to be normally distributed with a mean of 0 and the variance ω²_E0_ fixed to the value obtained for each parameter estimate of equation 1 (Table 1).

An impact of mucin on PD model parameters was tested on top of PMB binding to mucin with the following equation:


(4) $$P_{j}=\theta_{j,1}\times \left(1-Mucin\right)+\theta_{j,2}\times Mucin,$$

where *P*_*j*_ was the *j*th parameter of the PD model, *Mucin* was a categorical variable either equal to 0 or 1 depending on its absence or presence in TK experiments respectively, θ_*j*,1_ and θ_*j*,2_ corresponded to the *P*_*j*_ value in the absence and in the presence of mucin, respectively.

Model selection was based on OFV, relative standard errors of the parameter estimates obtained using the sampling importance resampling procedure implemented in PsN (42) and GOF plots (43). VPCs with stratification on PMB concentration and mucin presence were drawn to evaluate the predictive performance of the model and taken into account for model selection. Observed bacterial counts were plotted versus time and overlaid with the median and 90% prediction interval obtained by simulating 1,000 replicates of the original data set. The concordance between simulations and observations was inspected visually.

## SUPPLEMENTAL MATERIAL

Supplemental figures aac.01535-24-s0001.docx Figures S1 to S5.

Supplemental text aac.01535-24-s0002.docx Details on LC-MS/MS procedure, PK/PD modeling, and sequencing.

Supplemental tables aac.01535-24-s0003.docx Tables S1 to S3.

ASM does not own the copyrights to Supplemental Material that may be linked to, or accessed through, an article. The authors have granted ASM a non-exclusive, world-wide license to publish the Supplemental Material files. Please contact the corresponding author directly for reuse.
