# Supplementary material for: In vitro pharmacokinetic/pharmacodynamic modeling of the effect of mucin on polymyxin B activity against Acinetobacter baumannii
Source: Antimicrob Agents Chemother. 2025 Mar 26;69(5):e01535-24. doi: 10.1128/aac.01535-24 (PMC12057341; doi:10.1128/aac.01535-24)
Supplement: Supplemental figures — Figures S1 to S5. [file aac.01535-24-s0001.docx]

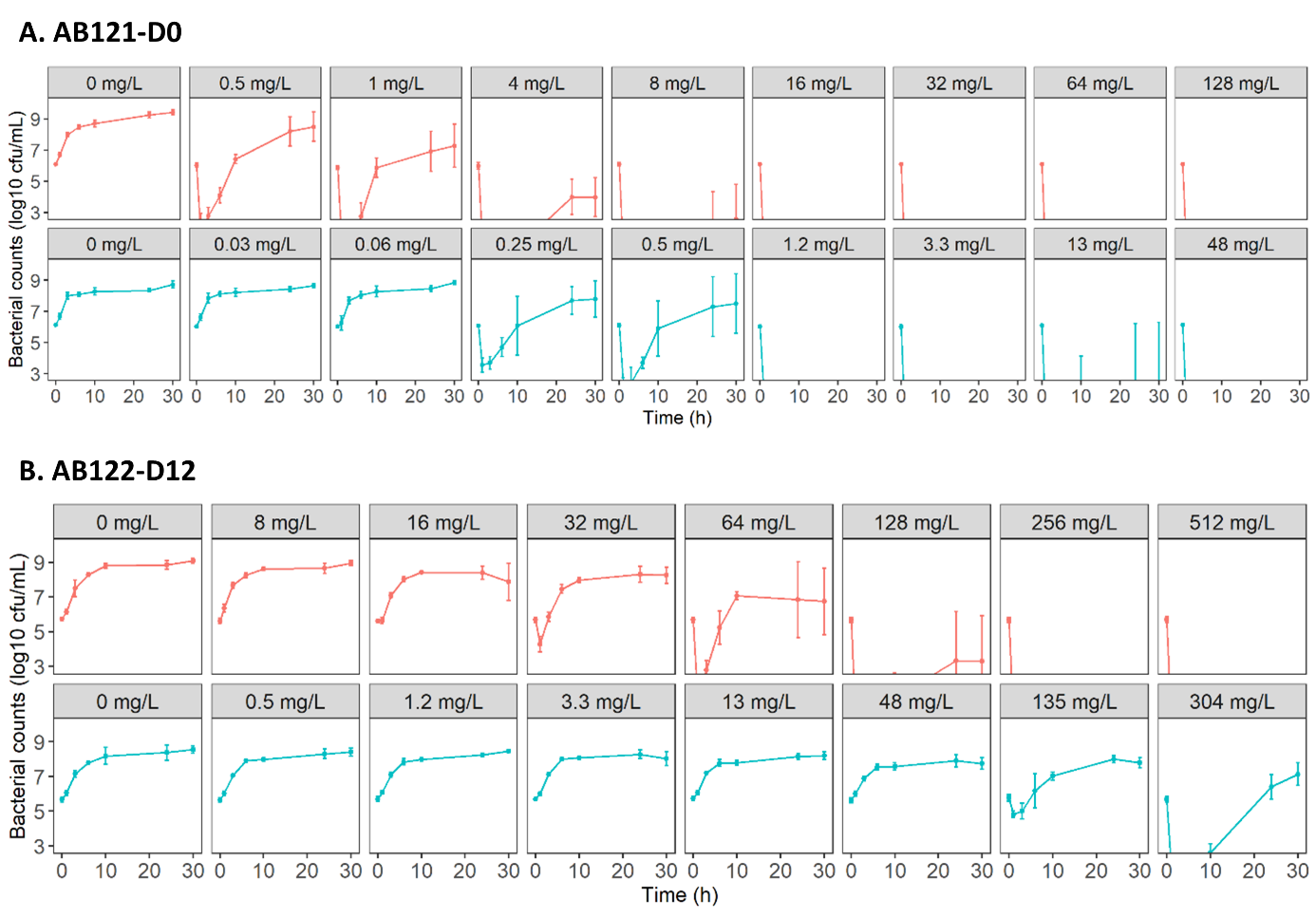


Figure S1: *Time-kill curves of AB121-D0 and AB122-D12. Polymyxin B concentrations are in mg/L. Lines correspond to the mean and error bars to the standard deviation of triplicates. Red: data without mucin. Turquoise: data with 1% mucin*


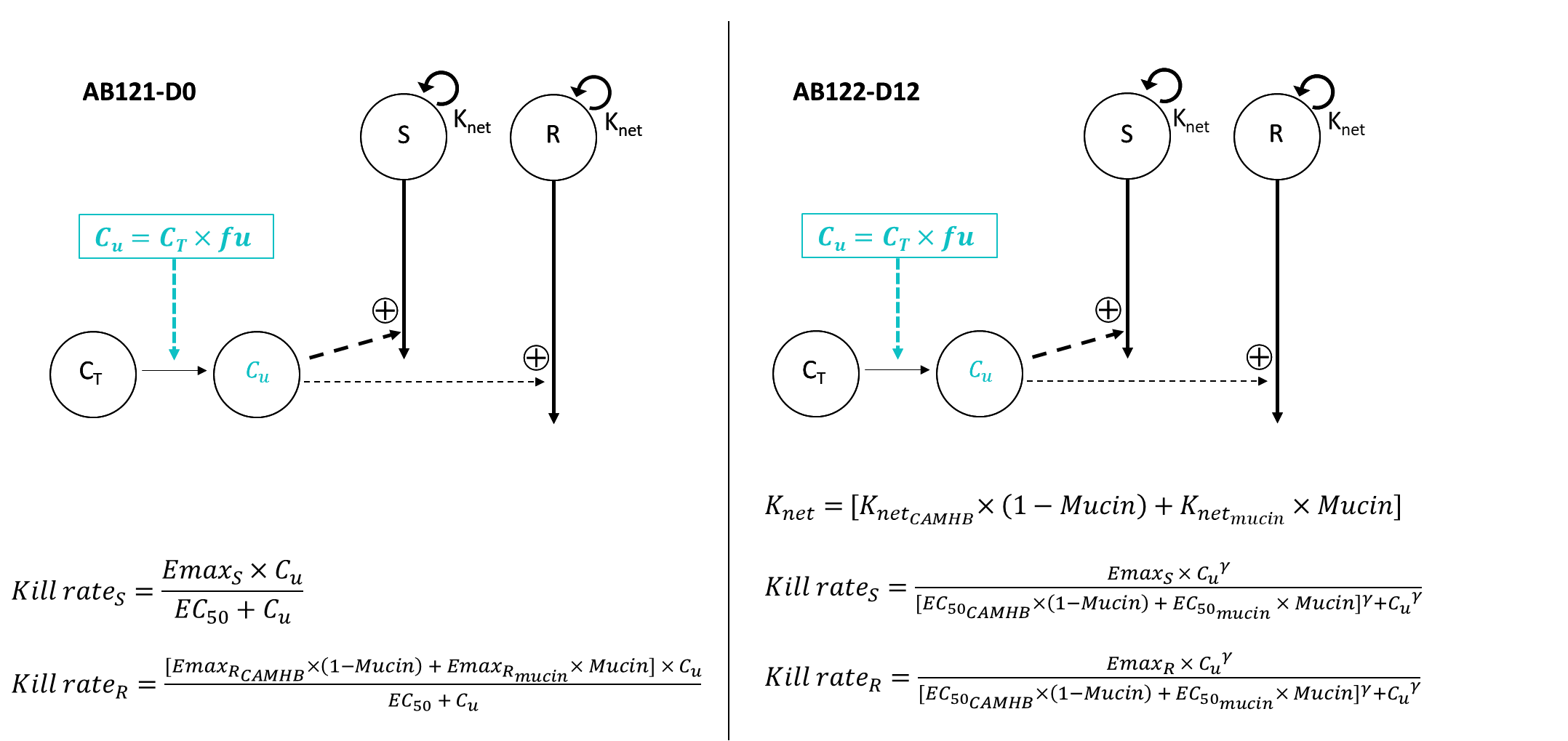


Figure S2: PK/PD models structure for both strains. K_net_: apparent growth rate constant (h^-1^); S: susceptible bacterial subpopulation; R: resistant bacterial subpopulation; C_T_ and C_u_: total and unbound polymyxin B concentrations (mg/L), respectively; Emax: maximum kill rate constant due to PMB (h^-1^); EC_50_: PMB concentration needed to reach 50% of Emax (mg/L); γ: Hill coefficient. Mucin is equal either to 0 or 1 regarding its absence or presence in TK experiment, respectively. Mucin had an impact on Emax_R_ for AB121-D0, and on K_net_ as well as on EC_50_ common to both bacterial subppulations for AB122-D12.


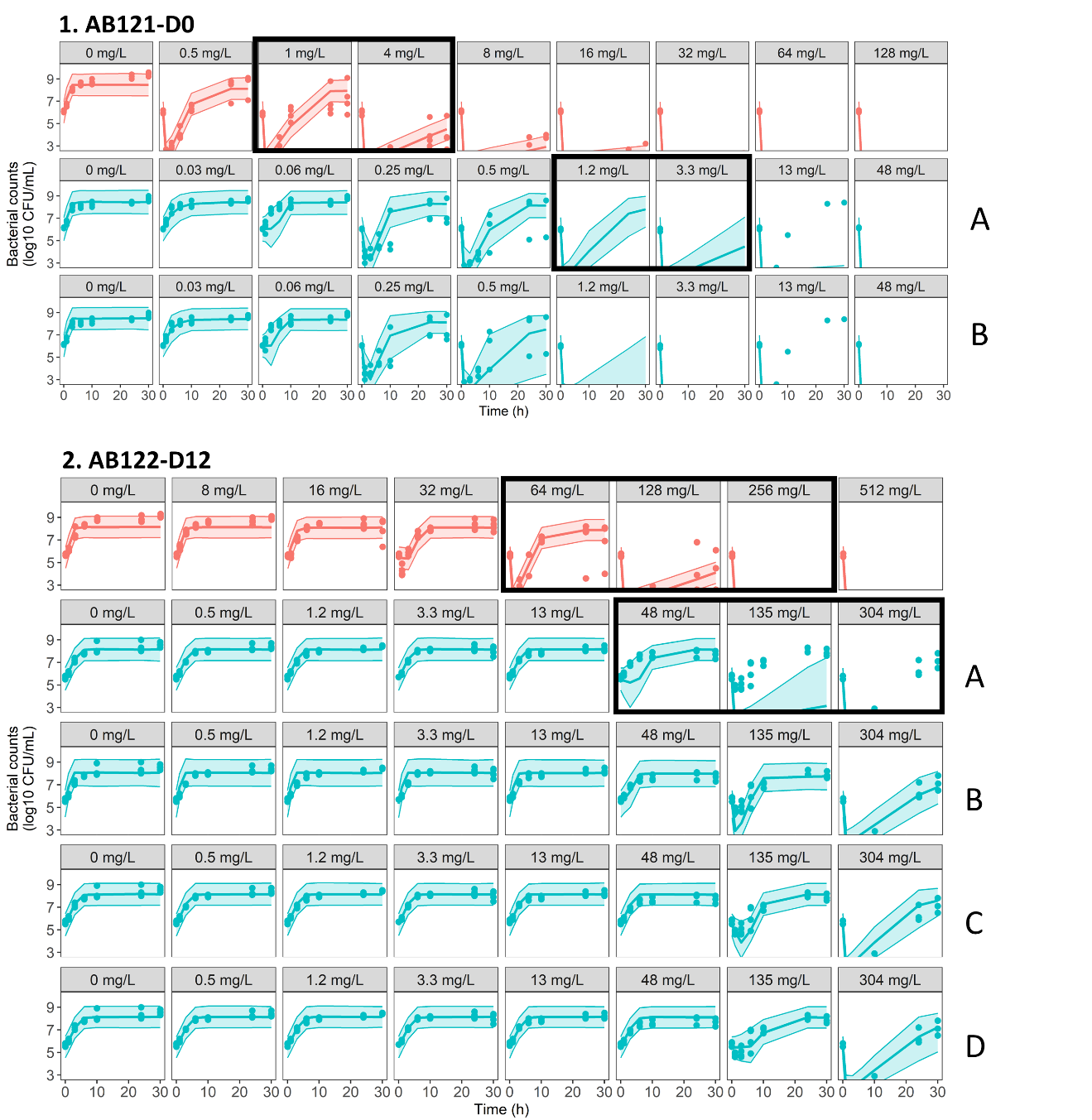


Figure S3: Visual predictive check (VPC) of final and intermediate models for AB121-D0 and AB122-D12. Circles represent experimental data, solid lines depict the median of simulated data, and colored areas depict the 90% prediction interval for 1,000 simulated profiles. Red: data without mucin. Turquoise: data with 1% mucin. For AB121-D0 : (A) VPC of model taking into account only the correction of PMB total concentrations with *fu* and no additional mucin effect (OFV = 263.254), and (B) final model taking into account the correction of PMB total concentrations with *fu* and a mucin effect on Emax_R_ (OFV= 111.29). For AB122-D12: (A) VPC of model taking into account only the correction of PMB total concentrations with *fu* and no additional mucin effect (OFV= 303.069), (B) model taking into account the correction of PMB total concentrations with *fu* and a mucin effect on K_net_ (OFV= 167.455), (C) model taking into account the correction of PMB total concentrations with *fu* and a mucin effect on EC_50_ (OFV= 23.884), and (D) final model taking into account the correction of PMB total concentrations with *fu* and a mucin effect on both K_net_ and EC_50_ (OFV = -4.007). The PMB concentrations indicated represent PMB unbound concentrations in TK experiments. Black boxes highlight the different CFU profiles obtained with and without mucin for similar concentrations of unbound PMB.


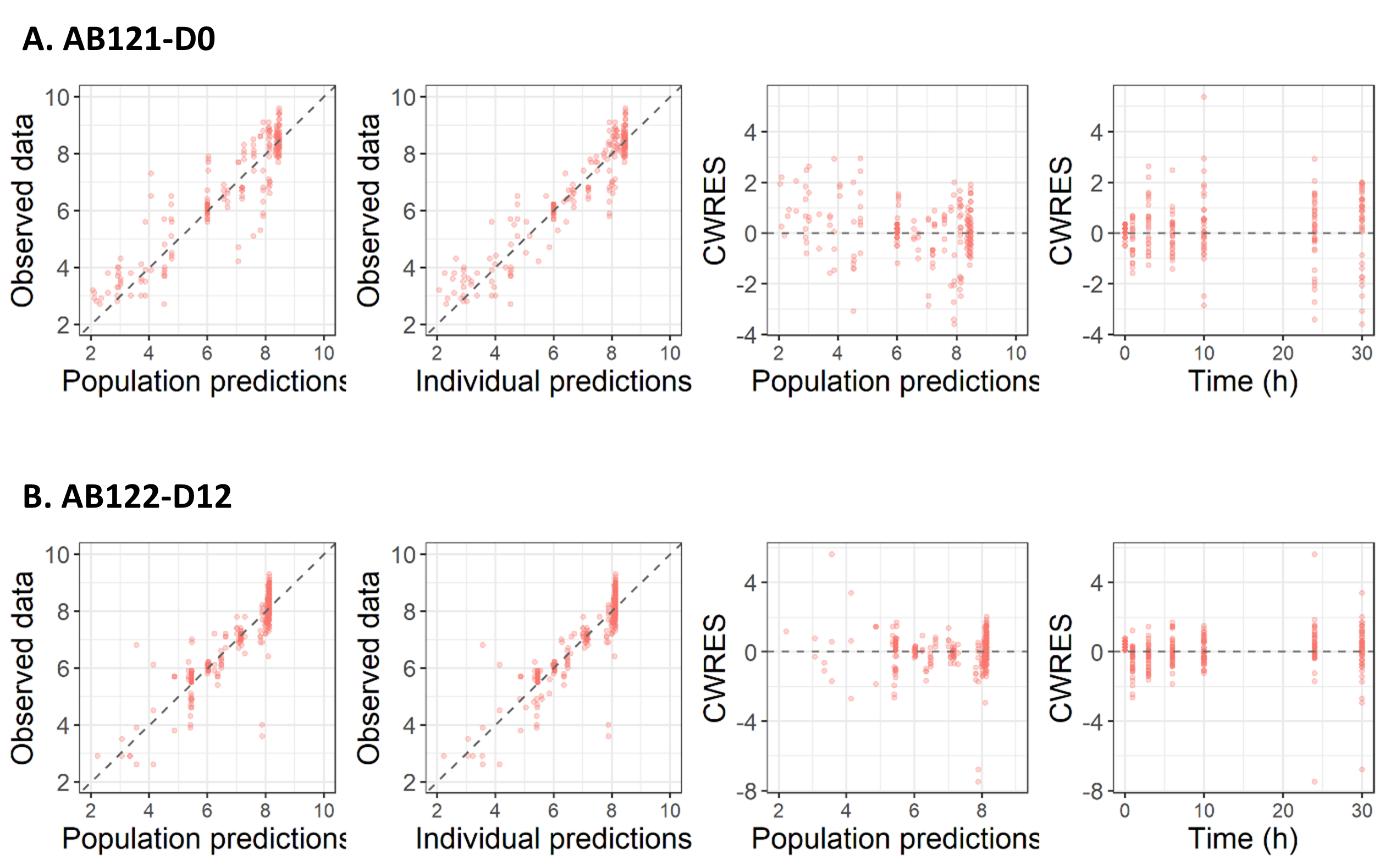


Figure S4: *Goodness of Fit plots (GOF) for AB121-D0 and AB122-D12*. CWRES, conditional weighted residuals.


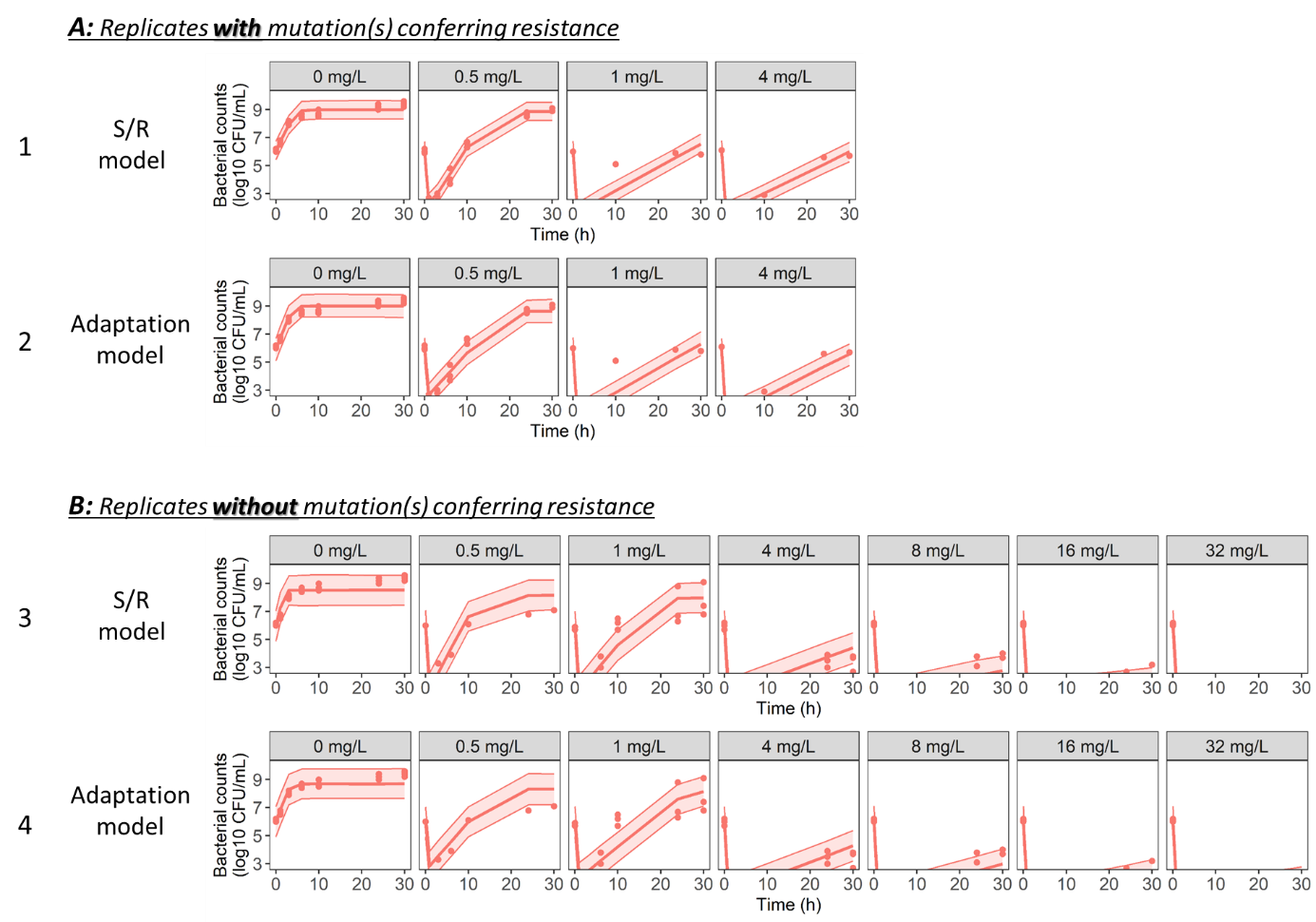


Figure S5: Visual predictive check of AB121-D0 TK data in CAMHB. Experimental replicates were separated regarding the presence (A) or not (B) of mutation(s) inducing PMB resistance, and modelled either by an S/R or an adaptation model. Circles represent experimental data, solid lines depict the median of simulated data and, colored areas depict the 90% prediction interval for 1,000 simulated profiles. AKAIKE values for 1, 2, 3 and 4 were respectively -21.3, 3.4, 53.3, and 54.7.
