# Supplementary material for: In vitro pharmacokinetic/pharmacodynamic modeling of the effect of mucin on polymyxin B activity against Acinetobacter baumannii
Source: Antimicrob Agents Chemother. 2025 Mar 26;69(5):e01535-24. doi: 10.1128/aac.01535-24 (PMC12057341; doi:10.1128/aac.01535-24)
Supplement: Supplemental text — Details on LC-MS/MS procedure, PK/PD modeling, and sequencing. [file aac.01535-24-s0002.docx]

# Supplemental material

## LC-MS/MS procedure

Briefly, 0.2 mL of sample added to 0.05 mL of drug-free plasma, were mixed with 0.75 mL of phosphate buffer (pH 7.2) and 0.02 mL of internal standard (colistin sulfate; Merck KGaA) at 6.25 mg/L. The samples were briefly vortexed and then centrifuged at 3000 rpm for 5 min. The supernatants (0.950 mL) were loaded onto SPE columns (Oasis HLB solid-phase extraction (SPE) cartridges (1 mL, 30 mg), Waters, Saint-Quentin-en-Yvelines, France), preconditioned with 1 mL of methanol followed by 1 mL of water. Then the columns were washed first with 1 mL of water and then with 1 mL of a solution of methanol/water (50/50). Columns were dried under a nitrogen pressure. The analytes were eluted with 0.5% formic acid in methanol. They were evaporated at 45°C under a gentle nitrogen jet stream. The residues were dissolved in 0.1 mL of 0.1% formic acid in water and analyzed by LC-MS/MS. The system included an Alliance Waters 2695 liquid chromatography system module (Waters) coupled with an API Quattro Micro (Waters). PMB was analyzed on an XBridge C_18_ column (5 µm, 2.1 x 150 mm; Waters). The mobile phase A consisted of 0.1% formic acid in water, and mobile phase B was 0.1% formic acid in acetonitrile. The gradient for mobile phase A and B were respectively set at 75 and 25% with a flow rate of 0.2 mL/min. Electrospray ionization in positive mode was used for the detection of PMB. Ions were analyzed in the multiple reaction monitoring, and the following transitions were inspected: *m/z* 602.1→101 for PMB1, *m/z* 595.1→101 for PMB2, and *m/z* 585.1→101 for colistin A and *m/z* 578.1→101 colistin B. Calibration curves of PMB ranged from 0.1 to 10 mg/L, and were quantified with a quadratic regression mode. The intraday variability was characterized at three levels (0.15, 1.5 and 7.5 mg/L) with a precision and bias of <20% for the lowest level, and < 15% for the others.

## PK/PD modelling

*Bacterial growth sub-model*

A previously described semi-mechanistic model with hetero-resistance (1), *i.e.* two bacterial sub-populations with S representing the susceptible and R the resistant bacterial sub-populations to PMB, was used to describe the time course of bacterial counts (CFU/mL) for the two *A. baumannii* strains. Briefly, a logistic growth model with one compartment for each bacterial sub-population was used to describe the self-limited bacterial growth in absence of antibiotic (Equations S1 and S2).

$\frac{dS}{dt}=k_{net}\times\left( 1-\frac{S+R}{BMAX} \right)\times S$ (Eq. S1)

$\frac{dR}{dt}=k_{net}\times\left( 1-\frac{S+R}{BMAX} \right)\times R$ (Eq. S2)

Where k_net_ (h^-1^) is the apparent growth rate constant and BMAX (CFU/mL) is the maximal capacity of the system.

*Drug effect sub-model*

PMB was assumed to have an effect on total bacteria.

$\frac{dS}{dt}=k_{net}\times\left( 1-\frac{S+R}{BMAX} \right)\times S-{Kill rate}_{S}\times S$ (Eq. S3)

$\frac{dR}{dt}=k_{net}\times\left( 1-\frac{S+R}{BMAX} \right)\times R-{Kill rate}_{R}\times R$ (Eq. S4)

Where Kill rate_S_ and Kill rate_R_ (h^-1^) are the killing rates of S and R respectively. Multiple functions for each kill rate were tested: a linear function ($Kill rate=Slope\times C$), a power function ($Kill rate=Slope\times C^{\gamma}$), an Emax function ($Kill rate=\frac{Emax \times C}{{EC}_{50} + C}$) or a sigmoidal Emax function ($Kill rate=\frac{Emax \times C^{\gamma}}{{{EC}_{50}}^{\gamma}+ C^{\gamma}}$). The final equations describing PMB kill rate for each strain and subpopulation were the following:

For AB121-D0

${Kill rate}_{S}=\frac{{Emax}_{S}\times C}{{EC}_{50}+ C}$ (Eq. S5)

${Kill rate}_{R}=\frac{{Emax}_{R}\times C}{{EC}_{50}+ C}$ (Eq. S6)

For AB122-D12

${Kill rate}_{S}=\frac{{Emax}_{S}\times C^{\gamma}}{{{EC}_{50}}^{\gamma}+ C^{\gamma}}$ (Eq. S7)

${Kill rate}_{R}=\frac{{Emax}_{R}\times C^{\gamma}}{{{EC}_{50}}^{\gamma}+ C^{\gamma}}$ (Eq. S8)

Where Emax (h^-1^) represents the maximum kill rate due to PMB, EC_50_ (mg/L) corresponds to PMB concentration needed to reach 50% of the maximum effect, C (mg/L) to the PMB concentration and γ to the Hill coefficient.

*Estimation and evaluation methods*

Model selection was based on objective function value (OFV) and goodness of fit plots. Visual predictive checks (VPCs) based on 1000 simulations were drawn to evaluate the fit to data and taken into account for model selection.

Bacterial count data were transformed into decimal logarithms before parameter estimation.

Data below the LOQ were taken into account in the model estimation by applying Beal’s M3 method (2). Uncertainty around population parameter estimates was estimated using the sampling importance resampling (SIR) technique. SIR options were –samples = 1000,1000,1000,2000 -resamples=200,400,500,1000 and results were considered final when the degrees of freedom (ddf) of SIR dOFV distribution reached the ddf of the theoretical dOFV distribution as described in (3).

Dataset preparation was performed using R software, parameter estimation was performed using NONMEM software (ICON, Dublin, Ireland) version 7.4.2 using the LAPLACIAN algorithm.

## Sequencing

Genomic DNA (gDNA) was extracted from overnight cultured using QIAamp PowerFecal Pro DNA Kit (Qiagen, Hilden, Germany) according to manufacturer’s instructions. Total extracted gDNA was quantified using a Nanodrop One (Thermofischer Scientific, Waltham, MA, USA). Long-read sequencing was performed during 24h using a MinION sequencer (Oxford Nanopore Technologies (ONT), Oxford, UK), ligation sequencing kit SQK-LSK109 (ONT) and flow cell FLO-MIN106 R9.4.1 (ONT) as previously described (4). Basecalling of raw data (FAST5 format) was performed using guppy (ONT; v5.0.17) in Super-Accurate mode (Qscore C 10). Filtlong (v0.2.1) was used to remove all short fragments (5). Genomes were assembled by using Trycycler (6) which combine the *de novo* assembler softwares (flye (v2.9-b1769), miniasm (v0.3-r179) + minipolish (v0.1.3) and raven (v1.8.1) (7–9)). Then, assembled genomes were polished with medaka (ONT; v1.5.0) using defaults settings of the medaka_consensus program (one round of polishing) (10). Median coverage was 100 for genomes and 130 for plasmids. Gene prediction was performed with the prokka pipeline (11) using *Acinetobacter baumannii* TCDC-AB0715 (GCA_000189735.2) transcript and protein datasets.

This Whole Genome Shotgun project has been deposited at DDBJ/ENA/GenBank under the number of PRJNA1120783. BioSample numbers are from SAMN41708040 to SAMN41708054.

# References

1. Mouton JW, Vinks AA, Punt NC. 1997. Pharmacokinetic-pharmacodynamic modeling of activity of ceftazidime during continuous and intermittent infusion. Antimicrob Agents Chemother 41:733–738.

2. Beal SL. 2001. Ways to Fit a PK Model with Some Data Below the Quantification Limit. J Pharmacokinet Pharmacodyn 28:481–504.

3. Dosne A-G, Bergstrand M, Karlsson MO. 2013. Application of Sampling Importance Resampling to estimate parameter uncertainty distributions. PAGE, Glasgow, Scotland.

4. Akrong G, Chauzy A, Aranzana-Climent V, Lacroix M, Deroche L, Prouvensier L, Buyck JM, Couet W, Marchand S. 2022. A New Pharmacokinetic-Pharmacodynamic Model To Characterize the Inoculum Effect of Acinetobacter baumannii on Polymyxin B In Vitro. Antimicrob Agents Chemother 66:e0178921.

5. Wick R. 2023. rrwick/Filtlong. C++.

6. Wick RR, Judd LM, Cerdeira LT, Hawkey J, Méric G, Vezina B, Wyres KL, Holt KE. 2021. Trycycler: consensus long-read assemblies for bacterial genomes. Genome Biology 22:266.

7. Vaser R, Šikić M. 2021. Time- and memory-efficient genome assembly with Raven. 5. Nat Comput Sci 1:332–336.

8. Kolmogorov M, Yuan J, Lin Y, Pevzner PA. 2019. Assembly of long, error-prone reads using repeat graphs. 5. Nat Biotechnol 37:540–546.

9. Wick RR, Holt KE. 2019. Benchmarking of long-read assemblers for prokaryote whole genome sequencing. F1000Res 8:2138.

10. 2024. nanoporetech/medaka. Python. Oxford Nanopore Technologies.

11. Seemann T. 2014. Prokka: rapid prokaryotic genome annotation. Bioinformatics 30:2068–2069.
