# Supplementary material for: In vitro pharmacokinetic/pharmacodynamic modeling of the effect of mucin on polymyxin B activity against Acinetobacter baumannii
Source: Antimicrob Agents Chemother. 2025 Mar 26;69(5):e01535-24. doi: 10.1128/aac.01535-24 (PMC12057341; doi:10.1128/aac.01535-24)
Supplement: Supplemental tables — Tables S1 to S3. [file aac.01535-24-s0003.docx]

| *Table S1: Parameter estimates of the Emax model for data generated with autoclaved or non-autoclaved mucin and all data pooled in a single dataset (Equation 1)* | | | |
| --- | --- | --- | --- |
| **Parameter** | **Pooled data** | **Autoclaved mucin** | **Non-autoclaved mucin** |
| **E0** | 0.061 | 0.067 | 0.056 |
| **Imax** | 0.56 | 0.56 | 0.57 |
| **IC_50_ (mg/L)** | 114.37 | 104.01 | 126.95 |
| **ε** | 1.97 | 2.21 | 1.87 |
| **R²** | 0.92 | 0.94 | 0.90 |

| *Table S2: fu (%) simulated using Equation 1 and parameters in Table S1. CI: Confidence Interval* | | | | | | |
| --- | --- | --- | --- | --- | --- | --- |
|  | **Pooled data** | | **Autoclaved mucin** | | **Non-autoclaved mucin** | |
| **Total conc.** | ***fu* [%] (95% CI)** | **Unbound conc.** | ***fu* [%] (95% CI)** | **Unbound conc.** | ***fu* [%] (95% CI)** | **Unbound conc.** |
| **0.5** | 6.1 (2.1 - 10.0) | 0.03 | 6.7 (0.1 - 13.2) | 0.03 | 5.6 (2.1 - 9.1) | 0.03 |
| **1** | 6.1 (2.1 - 10.0) | 0.06 | 6.7 (0.1 - 13.2) | 0.07 | 5.6 (2.1 - 9.1) | 0.06 |
| **2** | 6.1 (2.2 - 10.0) | 0.12 | 6.7 (0.1 - 13.2) | 0.13 | 5.6 (2.2 - 9.1) | 0.11 |
| **4** | 6.1 (2.3 - 10.0) | 0.25 | 6.7 (0.3 - 13.2) | 0.27 | 5.7 (2.3 - 9.1) | 0.23 |
| **8** | 6.4 (2.7 - 10.0) | 0.51 | 6.9 (0.7 - 13.0) | 0.55 | 5.9 (2.7 - 9.1) | 0.48 |
| **10** | 6.5 (3.0 - 10.1) | 0.65 | 7.0 (1.0 - 13.0) | 0.70 | 6.1 (3.0 - 9.2) | 0.61 |
| **50** | 15.2 (10.7 - 19.7) | 7.62 | 15.9 (7.7 – 24.2) | 7.96 | 14.2 (10.5 – 17.8) | 7.08 |
| **100** | 30.4 (25.6 - 35.2) | 30.4 | 33.4 (24.9 – 41.9) | 33.4 | 27.9 (23.8 – 32.1) | 27.9 |
| **500** | 59.2 (55.1 - 63.2) | 296 | 60.8 (55.0 - 66.6) | 304 | 58.6 (54.6 - 62.6) | 293 |
| **1000** | 61.3 (58.2 - 64.4) | 613 | 62.1 (57.2 – 67.1) | 621 | 61.5 (58.4 – 64.7) | 615 |
| **5000** | 62.1 (58.4 - 65.8) | 3100 | 62.5 (57.1 – 67.9) | 3120 | 62.7 (58.6 – 66.7) | 3130 |
| **10000** | 62.1 (58.3 - 65.8) | 6210 | 62.5 (57.1 – 68.0) | 6250 | 62.7 (58.6 - 66.9) | 6270 |

*Table S3: Accession numbers and links of bacterial samples used in this study and referenced on the National Center for Biotechnology Information (NCBI)*

| **Accession** | **Organism** | **Sample** | **Parental strain** | **Link** |
| --- | --- | --- | --- | --- |
| SAMN41708040 | *Acinetobacter baumannii* | AB121-PMB-0.5mg/L-w/o_mucin-Rep2 | AB121-D0 | https://www.ncbi.nlm.nih.gov/biosample/41708040 |
| SAMN41708041 | *Acinetobacter baumannii* | AB121-PMB-0.5mg/L-w/o_mucin-Rep3 | AB121-D0 | https://www.ncbi.nlm.nih.gov/biosample/41708041 |
| SAMN41708042 | *Acinetobacter baumannii* | AB121-PMB-0.5mg/L-w/o_mucin-Rep4 | AB121-D0 | https://www.ncbi.nlm.nih.gov/biosample/41708042 |
| SAMN41708043 | *Acinetobacter baumannii* | AB121-PMB-1mg/L-w/o_mucin-Rep4 | AB121-D0 | https://www.ncbi.nlm.nih.gov/biosample/41708043 |
| SAMN41708044 | *Acinetobacter baumannii* | AB121-PMB-4mg/L-w/o_mucin-Rep2 | AB121-D0 | https://www.ncbi.nlm.nih.gov/biosample/41708044 |
| SAMN41708045 | *Acinetobacter baumannii* | AB121-PMB-0.25mg/L-1%_mucin-Rep3 | AB121-D0 | https://www.ncbi.nlm.nih.gov/biosample/41708045 |
| SAMN41708046 | *Acinetobacter baumannii* | AB121-PMB-0.5mg/L-1%_mucin-Rep2 | AB121-D0 | https://www.ncbi.nlm.nih.gov/biosample/41708046 |
| SAMN41708047 | *Acinetobacter baumannii* | AB121-PMB-0.5mg/L-1%_mucin-Rep3 | AB121-D0 | https://www.ncbi.nlm.nih.gov/biosample/41708047 |
| SAMN41708048 | *Acinetobacter baumannii* | AB121-PMB-13mg/L-1%_mucin-Rep4 | AB121-D0 | https://www.ncbi.nlm.nih.gov/biosample/41708048 |
| SAMN41708049 | *Acinetobacter baumannii* | AB122-PMB-16mg/L-w/o_mucin-Rep1 | AB122-D12 | https://www.ncbi.nlm.nih.gov/biosample/41708049 |
| SAMN41708050 | *Acinetobacter baumannii* | AB122-PMB-128mg/L-w/o_mucin-Rep1 | AB122-D12 | https://www.ncbi.nlm.nih.gov/biosample/41708050 |
| SAMN41708051 | *Acinetobacter baumannii* | AB122-PMB-128mg/L-w/o_mucin-Rep2 | AB122-D12 | https://www.ncbi.nlm.nih.gov/biosample/41708051 |
| SAMN41708052 | *Acinetobacter baumannii* | AB122-PMB-48mg/L-1%_mucin-Rep2 | AB122-D12 | https://www.ncbi.nlm.nih.gov/biosample/41708052 |
| SAMN41708053 | *Acinetobacter baumannii* | AB122-PMB-135mg/L-1%_mucin-Rep3 | AB122-D12 | https://www.ncbi.nlm.nih.gov/biosample/41708053 |
| SAMN41708054 | *Acinetobacter baumannii* | AB122-PMB-135mg/L-1%_mucin-Rep4 | AB122-D12 | https://www.ncbi.nlm.nih.gov/biosample/41708054 |
